# Confirmatory factor analysis of the internet addiction test in university students

**DOI:** 10.15649/cuidarte.4593

**Published:** 2025-08-22

**Authors:** Manuela de Mendonça Figueirêdo Coelho, Viviane Mamede Vasconcelos Cavalcante, Tifanny Horta Castro, Mônica Oliveira Batista Oriá, Eveline Pinheiro Beserra, Fabiane do Amaral Gubert, Mariana Cavalcante Martins, Marília Braga Marques, Paula Sacha Frota Nogueir, Janaína Fonseca Victor Coutinho, Rachel Gabriel Bastos Barbosa

**Affiliations:** 1 Professor at the Federal University of Ceará, UFC. Fortaleza, Ceará, Brazil. E-mail: manumfc2003@yahoo.com.br UFC Ceará Brazil manumfc2003@yahoo.com.br; 2 Professor at the Federal University of Ceará, UFC. Fortaleza, Ceará, Brazil. E-mail: manumfc2003@yahoo.com.br UFC Ceará Brazil manumfc2003@yahoo.com.br; 3 Federal University of Ceará, UFC. Fortaleza, Ceará, Brazil. E-mail: tifannyhortacastro@gmail.com UFC Ceará Brazil tifannyhortacastro@gmail.com; 4 Professor at the Federal University of Ceará, UFC. Fortaleza, Ceará, Brazil. E-mail: monica.oria@ufc.br UFC Ceará Brazil monica.oria@ufc.br; 5 Professor at the Federal University of Ceará, UFC. Fortaleza, Ceará, Brazil. E-mail: eve_pinheiro@yahoo.com.br UFC Ceará Brazil eve_pinheiro@yahoo.com.br; 6 Professor at the Federal University of Ceará, UFC. Fortaleza, Ceará, Brazil. E-mail: fabianegubert@hotmail.com UFC Ceará Brazil fabianegubert@hotmail.com; 7 Professor at the Federal University of Ceará, UFC. Fortaleza, Ceará, Brazil. E-mail: marianaenfermagem@hotmail.com UFC Ceará Brazil marianaenfermagem@hotmail.com; 8 Professor at the Federal University of Ceará, UFC. Fortaleza, Ceará, Brazil. E-mail: mariliabm1@yahoo.com.br UFC Ceará Brazil mariliabm1@yahoo.com.br; 9 Professor at the Federal University of Ceará, UFC. Fortaleza, Ceará, Brazil. E-mail: sachanogueiraufc@gmail.com UFC Ceará Brazil sachanogueiraufc@gmail.com; 10 Professor at the Federal University of Ceará, UFC. Fortaleza, Ceará, Brazil. E-mail: janaina.victor@gmail.com UFC Ceará Brazil janaina.victor@gmail.com; 11 Professor at the Federal University of Ceará, UFC. Fortaleza, Ceará, Brazil. E-mail: rachelgabrielb@hotmail.com UFC Ceará Brazil rachelgabrielb@hotmail.com

**Keywords:** Factor Analysis, Internet Addiction, Students, Psychiatric Nursing, Mental Health, Análisis Factorial, Adicción a Internet, Estudiantes, Enfermería Psiquiátrica, Salud Mental, Análise Fatorial, Vício em Internet, Estudantes, Enfermagem Psiquiátrica, Saúde Mental

## Abstract

**Introduction:**

The Internet Addiction Test is a psychometric instrument designed to assess and quantify the severity of internet addiction and explore aspects of internet misuse.

**Objective:**

To confirm the factorial validity of the Internet Addiction Test in a sample of university students.

**Materials and Methods:**

A methodological study was conducted with 5,292 Brazilian university students. The data underwent confirmatory factor analysis and internal consistency assessment.

**Results:**

The Kaiser-Meyer-Olkin test yielded a value of 0.946, indicating that the sample was adequate. Factor analysis confirmed the existence of factors that fully align with the original American scale. The model showed satisfactory fit indices, including a Comparative Fit Index of 0.978, Tucker-Lewis Index of 0.976, Standardized Root Mean Residual of 0.053, and Root Mean Square Error of Approximation of 0.054 [0.052–0.056], which are consistent with previously established satisfactory fit criteria. Factor loadings of the items were considered high, ranging from 0.43 (item I7) to 0.89 (item I17).

**Discussion:**

The Internet Addiction Test’s six-factor structure was validated, which is identical to that proposed by the original American version and was consistent with other studies that have also identified the same multifactorial structure of the original instrument. Therefore, psychiatric nurses can confidently use it when caring for young university students.

**Conclusion:**

The Internet Addiction Test is a valid and reliable instrument for measuring internet addiction among university students and can be used by mental health nurses to identify such issues.

## Resumen

**Introducción:**

El Internet Addiction Test es un instrumento psicométrico diseñado para evaluar y cuantificar la gravedad de la adicción a Internet y explorar aspectos de su uso problemático.

**Objetivo:**

Confirmar la validez factorial del Internet Addiction Test en una muestra de estudiantes universitarios.

**Materiales y métodos:**

Se realizó un estudio metodológico con 5.292 estudiantes universitarios brasileños. Los datos fueron sometidos a un análisis factorial confirmatorio y de consistencia interna.

**Resultados:**

Los resultados del test de Kaiser-Meyer-Olkin arrojó un valor de 0,946, lo cual indica que la muestra era adecuada. El análisis factorial confirmó la existencia de factores que se alineaban completamente con la escala estadounidense original. El modelo mostró índices de ajuste satisfactorios, incluyendo un índice de ajuste comparativo de 0,978, índice de Tucker-Lewis de 0,976, residuo cuadrático medio estandarizado de 0,053 y error cuadrático medio de aproximación de 0,054 [0,052-0,056], los cuales son consistentes con los criterios de ajuste satisfactorio establecidos previamente. Las cargas factoriales de los ítems se consideraron altas y oscilaron entre 0,43 (ítem I7) y 0,89 (ítem I17).

**Discusión:**

La estructura de seis factores del Internet Addiction Test fue validada y resultó idéntica a la propuesta por la versión estadounidense original y es consistente con otros estudios que también han identificado la misma estructura multifactorial del instrumento original. Por lo tanto, las enfermeras psiquiátricas pueden utilizarlo con confianza al atender a jóvenes universitarios.

**Conclusiones:**

El Internet Addiction Test es un instrumento válido y confiable para medir la adicción a Internet en estudiantes universitarios y puede ser utilizado por enfermeras de salud mental para identificar dichos problemas.

## Resumo

**Introdução:**

O Internet Addiction Test é um instrumento psicométrico desenvolvido para avaliar e quantificar a gravidade da dependência de Internet e explorar aspectos do uso indevido da Internet.

**Objetivo:**

Confirmar a validade fatorial do Internet Addiction Test em uma amostra de estudantes universitários.

**Materiais e métodos:**

Foi realizado um estudo metodológico com 5.292 estudantes universitários brasileiros, submetidos à análise fatorial confirmatória.

**Resultados:**

Os resultados do teste Kaiser-Meyer-Olkin (0,946) indicaram que a amostra era adequada. A análise fatorial confirmou a existência de fatores que não se alinhavam totalmente com a escala americana original. O modelo apresentou índices de ajuste satisfatórios, incluindo um Índice de Ajuste Comparativo de 0,978, Índice de Tucker-Lewis de 0,976, Raiz Média Residual Padronizada de 0,053 e Raiz Média Quadrática de Erro de Aproximação de 0,054 [0,052–0,056], que são consistentes com os critérios de ajuste satisfatórios previamente estabelecidos. As cargas fatoriais dos itens foram consideradas altas, variando de 0,43 (item I7) a 0,89 (item I17).

**Discussão:**

O modelo teórico do Internet Addiction Test foi validado em termos da composição de seis fatores, que é idêntico ao proposto pela versão americana original e é consistente com outros estudos que também identificaram a mesma estrutura multifatorial do instrumento original. Portanto, enfermeiros psiquiátricos podem usá-lo com confiança ao cuidar de jovens estudantes universitários.

**Conclusões:**

O Internet Addiction Test foi considerado um instrumento válido e confiável para medir o vício em Internet em estudantes universitários e pode ser utilizado por enfermeiros de saúde mental para identificar tais problemas.

## Introduction

University students' mental health cannot be overlooked any longer. It is imperative that anxiety, sleep disturbances, substance abuse, and other mental disorders be considered and included in discussions of public education and health policies^1^-^4^.

Internet overuse has been associated with adverse effects on individuals' health and well-being. For example, it is reasonable to infer that teenagers may exhibit high levels of stress, anxiety, and depression proportional to their levels of internet addiction^5^. Another significant consequence of pervasive internet use is the tendency among university students to procrastinate academic activities^6^. Many students struggle to exercise self-control regarding the use of this technology^7^.

The Internet Addiction Test (IAT) is a psychometric instrument designed to assess and quantify the severity of internet addiction. It is a self-administered, publicly available instrument used to estimate an individual's level of internet addiction^8^. Although a Portuguese version was translated and validated in 2012^9^, this scale is not widely used in Brazil.

The IAT consists of 20 items grouped into six factors: salience, excessive use, neglect of work, anticipation, lack of control, and neglect of social life. The results are interpreted across three levels of usage: mild, moderate, and severe, with scores ranging from 0 to 100 points^9^,^10^. 

The salience factor refers to the persistent preoccupation with being connected to the internet. Excessive use is defined as a loss of time perception or the neglect of basic needs resulting from internet use. Neglect of work and social activities occurs when individuals cease to engage in these activities to remain online. Anticipation is defined as the act of using the internet even before addressing any other important needs. The concept of lack refers to the user's inability to regulate the amount of time spent online^8^.

The internal consistency of the IAT has been evaluated in several studies conducted worldwide. In the United Kingdom, factor analysis confirmed its internal consistency and corroborated the six-factor structure. Consistency coefficients ranged from 0.54 to 0.82 across domains, with positive correlation among them^11^. Two additional studies, conducted in Italy and Switzerland, also identified the presence of six factors, with one factor having a prominent influence on the scale's overall variance^12^,^13^.

In Brazil, the initial internal consistency analysis of the instrument demonstrated satisfactory internal consistency for most items (items 01 and 07 exhibited suboptimal results). Moreover, the six-factor structure was also confirmed; however, the grouping of items only partially aligned with the original scale's distribution^10^. Given the limited sample size used in this study, further evaluations of the factor structure were recommended. In 2021, a new psychometric evaluation of the scale was conducted in Brazil, identifying the presence of only three factors^14^.

Nurses working in mental health should pay attention to this type of addiction that has been developing among young people, and conduct assessments for signs and symptoms of internet addiction, bearing in mind that it can have a negative impact on individuals' lives, especially since it has been associated with high levels of depression and negative body image^15^,^16^. Thus, with a reliable instrument, nurses will be able to objectively assess such behaviors and design nursing interventions in collaboration with the multidisciplinary team to minimize or mitigate this condition.

In light of the above, it is imperative to present how the factor structure of the IAT manifests in this specific sample to contribute evidence regarding the consistency of the instrument and assess whether it maintains its original factorial structure. This is particularly important given that the IAT has already undergone factor analysis and is used to measure the degree of internet dependency.

Considering the discrepancies found in previous studies, particularly those conducted in Brazil, it is imperative to conduct a confirmatory factor analysis of the IAT. The objective of this study is to confirm the factorial validity of the IAT in a sample of Brazilian university students.

## Materials and Methods

The study employed a cross-sectional design with a quantitative approach. The study sample consisted of Brazilian university students enrolled in public (federal and state) and private higher education institutions. The inclusion criteria required participants to be over 18 years and to have completed at least the first semester of their undergraduate or graduate program. Incomplete forms were excluded from the analysis. 

For sample size calculation, the census from the Brazilian Ministry of Education was considered, which indicates that approximately two million people are enrolled in universities in Brazil. Thus, the research sample was calculated considering a 95% confidence level and a 3% margin of error, resulting in a minimum required sample size of 3,198 participants. However, the objective was to collect as much data as possible to increase the generalizability of the results. Consequently, of the 5,345 responses received, only 5,292 were included in the final sample. The STROBE guidelines, as recommended by the EQUATOR Network for observational studies, were followed. Data from this survey are available on the OSFHome platform^17^.

To collect data, an online questionnaire was employed, which included items on sociodemographic and academic characteristics, health conditions, and the Internet Addiction Test (IAT), composed of 20 Likert-scale items. These items are distributed across six factors: salience (items 10, 12, 13, 15, 19), excessive use (items 1, 2, 14, 18, 20), neglect of work (items 6, 8, 9), anticipation (items 7, 11), lack of control (items 5, 16, 17), and neglect of social life (items 3, 4).

Data collection took place online between November 2021 and March 2022. An initial survey of all public (federal and state) and private universities listed on the Brazilian Ministry of Education website was conducted. Subsequently, institutional websites were searched, and a survey of the courses offered by all universities with websites was conducted. The email addresses of the coordinators of all courses were also collected.

Subsequently, an invitation email was sent to these course coordinators, explaining the objective of the research and requesting that the invitation be forwarded to all enrolled students. This allowed students to decide freely whether to participate. The email also included the informed consent form, which could be digitally signed by those who wished to participate, as well as the data collection instruments.

The psychometric sensitivity of the IAT items was estimated using descriptive statistics, including central tendency, dispersion, kurtosis, and skewness. Items with skewness values below three and kurtosis values below seven were considered to have adequate sensitivity^18^.

The internal consistency of each factor was assessed using Cronbach's alpha coefficient (α), with α values of 0.70 or higher deemed appropriate. Sample adequacy was tested using the Kaiser-Meyer-Olkin (KMO) index and Bartlett's test of sphericity. A KMO index >0.5 and Bartlett's test of sphericity with p-value < 0.005 were considered adequate.

To evaluate inter-factor correlations, the Spearman correlation test was employed, with the following interpretation: Correlation coefficients ranging from 0.8 to 1.0 indicate a strong correlation, while coefficients between 0.5 and 0.8 indicate a moderate correlation. Coefficients of 0.2 to 0.5 indicate a weak correlation, while coefficients of 0.0 to 0.2 indicate negligible correlation.

A confirmatory factor analysis (CFA) was conducted to assess the plausibility of the IAT's multidimensional structure. The analysis was conducted using the robust diagonally weighted least squares (RDWLS) estimation method^19^,^20^.

Model fit was evaluated using the following indices: χ2, χ2/df, Comparative Fit Index (CFI), Tucker-Lewis Index (TLI), Standardized Root Mean Residual (SRMR), and Root Mean Square Error of Approximation (RMSEA). It is important to note that non-significant χ2 values are preferred. Furthermore, a χ2/df ratio should be less than 5, or preferably less than 3. Additionally, the Comparative Fit Index (CFI) and the Tucker-Lewis Index (TLI) values should be greater than 0.90, with a preference for values above 0.95. Finally, the Root Mean Square Error of Approximation (RMSEA) should be less than 0.08, or preferably less than 0.06, with a confidence interval (upper limit) less than 0.10^21^. Adequate factor loadings above 0.40 will be considered^22^.

The results were organized in an Excel spreadsheet and exported to the Statistical Package for the Social Sciences (SPSS), version 23.0, for descriptive, inferential, and correlational analyses. JASP software was employed for the factor analyses.

This study followed all the ethical principles outlined in the Declaration of Helsinki. Its content and execution were approved by the Research Ethics Committee of the Federal University of Ceará. All participants signed the informed consent form agreeing to participate in the study. The study was approved by the Research Ethics Committee under opinion number 4.277.440.

## Results

The sample included 5,292 university students from Brazil, with an average age of 24 (SD ± 6.9). Table 1 shows the students' sociodemographic, academic, and health-related data. 

**Table 1:** Sociodemographic, academic, behavioral, and health characteristics

Variables	% (n)
Sociodemographic	
Sex	
Female	67.36 (3565)
Male	32.64 (1727)
Race	
White	45.50 (2408)
Mixed race	40.28 (2132)
Black	11.70 (619)
Asian	1.78 (94)
Indigenous	0.74 (39)
Marital status	
Single	86.48 (4577)
Married/in a stable union	13.49 (713)
Widowed	0.03 (02)
Family income	
< 1 minimum wage	14.22 (753)
1 minimum wage	23.03 (1219)
From 1 to 5 minimum wages	49.50 (2619)
> 5 minimum wages	13.25 (701)
Region	
Northeast	61.98 (3280)
Southeast	12.30 (651)
North	11.60 (614)
Midwest	7.70 (407)
South	6.42 (340)
Academic	
Field of Study	
Human Sciences	20.80 (1101)
Exact and Earth Sciences	15.18 (803)
Health Sciences	15.04 (796)
Applied Social Sciences	14.38 (761)
Linguistics, Letters and Arts	13.39 (709)
Engineering	11.47 (607)
Biological Sciences	6.16 (326)
Agricultural Sciences	3.58 (189)
Type of Educational Institution	
Federal Public Institution	57.94 (3066)
State Public Institution	38.56 (2041)
Private Institution	3.50 (185)
Has completed another degree	
No	89.62 (4743)
Yes	10.38 (549)
Behavioral and health conditions	
Practiced social isolation during the pandemic	
Yes	85.36 (4517)
No	14.64 (775)
Tested positive for COVID-19	
Yes	34.12 (1806)
No	65.88 (3486)
Consumes alcoholic beverages	
Yes	48.20 (2551)
No	51.80 (2741)
Smokes	
Yes	8.60 (454)
No	91.40 (4839)
Consumes illicit drugs	
Yes	7.72 (409)
No	92.28 (4883)
Uses medication for depression	
Yes	13.35 (707)
No	86.65 (4585)
Uses medication for anxiety	
Yes	22.40 (1185)
No	77.60 (4107)
Receives psychotherapy	
Yes	21.56 (1141)
No	78.44 (4151)

The correlations among the IAT factors are shown in Table 2. 

**Table 2:** Correlation coefficients between the factors of the Internet Addiction Test

	Factor 1 Salience	Factor 2 Excessive use	Factor 3 Neglect of work	Factor 4 Anticipation	Factor 5 Lack of control	Factor 6 Neglect of social life
Factor 1 Salience	1	0.747*	0.534*	0.698*	0.666*	0.506*
Factor 2 Excessive use	-	1	0.569*	0.697*	0.778*	0.467*
Factor 3 Neglect of work	-	-	1	0.595*	0.519*	0.379*
Factor 4 Anticipation	-	-	-	1	0.654*	0.418*
Factor 5 Lack of control	-	-	-	-	1	0.411*

Spearman's rho coefficients. *p<0.001

A significant correlation was observed between salience and excessive use (rho=0.747), as well as between excessive use and a lack of control (rho=0.778). These values approach the threshold for a strong correlation. 

Psychometric sensitivity of the IAT items is reported in Table 3 through descriptive statistics. The flattening (kurtosis) values were not statistically significant, with small variations from 1.546 to 1.479. 

The Cronbach's α for the salience factor was α=0.779, for excessive use was α=0.771, for neglect of work was α=0.497, for anticipation was α=0.563, for lack of control was α=0.718, and for neglect of social life was α=0.291. The internal consistency of the IAT was deemed adequate (α=0.916), with a KMO index of 0.946 and a Bartlett's test of sphericity (p<0.001), indicating that the data were suitable for factor analysis. 

**Figure 1:**
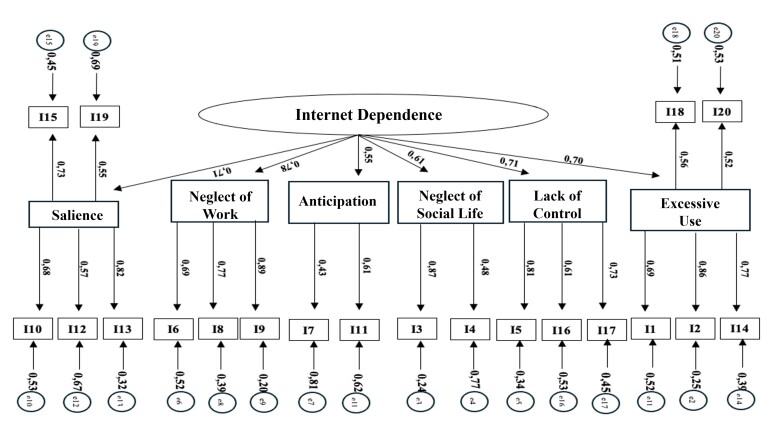
Confirmatory factor analysis for the Internet Addiction Test adjusted for a sample of Brazilian university students (n=5262)

A confirmatory factor analysis was conducted to assess the plausibility of the IAT's multidimensional structure. The model demonstrated good fit, with a χ2 value of 2766.580 (df = 170, p < 0.001). The χ2/df ratio for degrees of freedom was high (16.27), with CFI=0.978, TLI=0.976, SRMR= 0.053, and RMSEA= 0.054 [0.0052-0.0056]. These results meet the commonly accepted criteria for good model fit. The factor loadings of the items were high, ranging from 0.43 (item I 7) to 0.89 (item I 17) (Figure 1). 

**Table 3:** Distribution measures for items of the Internet Addiction Test among Brazilian university students (n=5292)

Item	Average Standard deviation
1) How often do you find that you stay online longer than you intended?	3.89 ±1.25
2) How often do you neglect household chores to spend more time online?	2.89 ±1.58
3) How often do you prefer the excitement of the Internet to intimacy/relationships with your partner/friends?	1.31 ±1.64
4) How often do you form new relationships with fellow online users?	1.78 ±1.67
5) How often do others in your life complain to you about the amount of time you spend online?	1.83 ±1.72
6) How often do your grades or school work suffer because of the amount of time you spend online?	1.57 ±1.72
7) How often do you check your email before something else that you need to do?	2.04 ±1.79
8) How often does your job performance or productivity suffer because of the Internet?	1.82 ±1.74
9) How often do you become defensive or secretive when anyone asks you what you do online?	1.22 ±1.64
10) How often do you block out disturbing thoughts about your life with soothing thoughts of the Internet?	2.16 ±1,93
11) How often do you find yourself anticipating when you will go online again?	1.64 ±1.75
12) How often do you feel that life without the Internet would be boring, empty, and joyless?	1.90 ±1.84
13) How often do you snap, yell, or act annoyed if someone bothers you while you are online?	0.96 ±1.46
14) How often do you lose sleep due to late-night logins?	2.05 ±1.84
15) How often do you feel preoccupied with the Internet when offline, or fantasize about being online?	1.36 ±1.65
16) How often do you find yourself saying “just a few more minutes” when online?	2.46 ±1.84
17) How often do you try to cut down the amount of time you spend online and fail?	2.29 ±1.79
18) How often do you try to hide how long you have been online?	1.18 ±1.67
19) How often do you choose to spend more time online over going out with others?	1.80 ±1.84
20) How often do you feel depressed, moody, or nervous when you are offline, which goes away once you are back online?	1.30 ±1.65

## Discussion

The results of the present study confirm that, within the analyzed sample, the theoretical model of the Internet Addiction Test (IAT), regarding its six-factor structure, is identical to the original American version^8^ and aligns with other studies that have also identified the same multifactorial structure as the original instrument^10^,^12^,^13^. The KMO test and Bartlett's test of sphericity indicated that the sample size was appropriate for factor analysis, with values above 0.50. Therefore, psychiatric nurses can confidently use it when caring for young university students.

A growing body of research is now focusing on the high rates of internet dependency observed in contemporary society^23^,^24^. The phenomenon of internet addiction among young people has been linked to adverse effects on mental health and the reinforcement of socially isolating behaviors^25^. Several problems have been associated with internet addiction among university students, including negative body image, alexithymia, reduced physical activity, communication difficulties, depression, and the use of psychoactive substances^26^,^27^. 

Nurses are already beginning to work to detect this problem. A study conducted by pediatric nurses in Turkey found that 89.4% of high school students participating in their study were dependent on smartphones and the internet^28^. In another study, nurses demonstrated that there is a negative correlation between internet addiction and students' emotional intelligence^29^.

Considering these findings, the use of valid and reliable instruments to measure internet addiction is of the utmost importance. Such instruments must be capable of accurately assessing the construct, thereby enabling the monitoring of internet addiction. This, in turn, enables health and education professionals, as well as family members, to implement prevention, control, and even treatment measures when necessary.

In this study, the internal consistency coefficients for the neglect of work factor (α=0.497), anticipation factor (α=0.563), and neglect of social life factor (α=0.291) were below the acceptable threshold. A psychometric study of the IAT conducted in Minas Gerais^14^ proposed readapt the items of the three aforementioned factors. In that study, items originally grouped under neglect of work, neglect of social life, and anticipation factors were reassigned to different factors. It is also important to consider that the low internal consistency observed may be related to the reduced number of items that comprise these factors.

Despite this, the present study identified an adequate factorial solution, satisfactory model fit, and acceptable psychometric sensitivity. All IAT items exhibited adequate levels of kurtosis and skewness, indicating that the score distribution approximated normality and that the instrument is capable of adequately discriminating between different evaluation levels.

Nevertheless, other studies have identified alternative factorial structures. A study conducted with medical students in Lebanon revealed a four-factor structure, which indicated the presence of lack of control, social withdrawal, emotional conflict, time management issues, and behavior concealment^30^. The psychometric evaluation of the Persian version of the IAT identified only two factors as necessary for the scale^31^. Similarly, an Indian study also confirmed the good internal consistency of the test based on a two-factor structure^32^. Despite the observed divergence in factor structures, appropriate factor loadings were observed in all items, with values exceeding 0.40^14^,^31^,^33^.

It is important to note that different factorial structures between the IAT items in different scenarios, populations, and cultural contexts highlight the importance of validating the IAT for diverse audiences. This underscores the importance of maintaining rigor in adapting the instrument in relation to the content, cultural reality, and heterogeneity of the samples studied.

Although internet addiction is not yet included in the Diagnostic and Statistical Manual of Mental Disorders (DSM-5), it is classified as a mental disorder in the International Classification of Diseases (ICD-11). Internet addiction is listed under the code 6C51, which refers to excessive use of digital devices, particularly involving online gaming^34^. Although there have been few studies conducted in Brazil using the IAT to identify the level of internet addiction^35^, it is imperative to draw attention to this condition.

It has been previously demonstrated that such dependence can be associated with suicidal ideation, unhealthy eating behaviors, and abnormal body weight, as well as impulsivity and aggression^15^,^36^,^37^. These findings suggest that such dependence may represent a significant yet largely overlooked public health concern.

The present study, which confirms the factorial validity of the IAT among university students, demonstrates that this instrument is a reliable and relevant tool for assessing internet addiction. Therefore, it should be incorporated into psychiatric nurses' care practices. 

With this in mind, attention is also drawn to the low number of psychiatric nursing studies in this area. Considering that the internet is an active part of students' lives, and thinking that this type of dependence may be associated with other disorders (stress and anxiety) as well as personal and academic dysfunction, it is urgent that psychiatric nurses are prepared to screen for this condition and implement early interventions^5^,^6^.

Thus, the greatest contribution of this study, in addition to confirming the structural validity of the IAT to measure this construct among university students, lies in rising awareness within psychiatric nursing about the problem so that we can incorporate the use of this instrument in our practice; however, how to foster interest and emphasize the importance of nursing care in addressing this addiction remains uncertain. The need for further studies in this area is also noted.

Finally, psychiatric nurses from all countries are encouraged to pursue cross-cultural psychometric validation of this instrument so that it can be widely used in psychiatric nursing worldwide, ensuring its linguistic and psychometric applicability.

**Strengths and limitations**

This study validates the IAT as an effective tool for assessing internet addiction levels among Brazilian university students, a crucial aspect in understanding the impact of internet use on mental health. The significance of this article for mental health nursing lies in the potential application of the IAT as a diagnostic and preventive tool. This allows nursing professionals to identify, intervene, and provide support to individuals with excessive internet use behaviors, thereby helping to minimize associated risks such as anxiety, depression, and other mental health disorders.

One limitation of the study is the absence of concurrent criterion validation. This step would have involved correlating the IAT with another instrument measuring the same construct and latent dimensions.

## Conclusions

The confirmatory analysis of the Internet Addiction Test (IAT) to assess levels of internet addiction among university students revealed that the Brazilian version of the instrument maintains a six-factor structure consistent with the original version, showed appropriate internal consistency, and can be used by psychiatric nurses or other professionals as a screening tool for internet addiction among university students. Consequently, it can be concluded that this instrument exhibits internal consistency and structural validity, making it suitable for assessing internet addiction in this context. It is recommended that researchers consider prior research and any new evidence when designing and conducting new studies involving the IAT. Psychiatric nursing, in particular, is encouraged to design and conduct research on this topic, including the transcultural and psychometric validation of the IAT worldwide.
